# A precise and high-throughput assay for stem structural characteristics deepens understanding of lodging resistance in sorghum

**DOI:** 10.1186/s12870-025-06396-y

**Published:** 2025-03-27

**Authors:** Jianguo Li, Liyan Zhao, Hongzeng Fan, Falin Zhao, Dandan He, Bo Li, Jibin Wang, Guosheng Xie, Zhen Hu, Chuchuan Fan, Lingqiang Wang

**Affiliations:** 1 State Key Laboratory for Conservation & Utilization of Subtropical Agro-Bioresources, College of Agriculture, Guangxi University, Nanning, China; 2 College of Plant Science & Technology, Huazhong Agricultural University, Wuhan, 430070 China; 3 Moutai Institute, Renhuai, Guizhou, 564507 China

**Keywords:** Sorghum, Stem lodging resistance, Visible/Near-infrared spectroscopy, Stem structural characteristics

## Abstract

**Background:**

Plant stem structural characteristics are crucial factors determining plant lodging resistance, while high throughput methods for rapid surveys of these traits are still lacking in sorghum.

**Results:**

Among 103 sorghum accessions, two kinds of stem powders (dry and water-washed) were subject to visible and near-infrared spectra acquisition, and 16 models (combinations) for stem structural characteristics were generated, revealing that the support vector machine regression model has significant positive effects on the prediction of stem structural characteristics while powder type and pretreatment of spectra has minor effects on the prediction of stem structural characteristics. In addition, we found that stem structure characteristics were positively correlated with agronomic traits but negatively correlated with lodging index which is the criterion that negatively accounts for plant lodging resistance.

**Conclusion:**

This study for the first time provided a precise and high throughput method for the prediction of sorghum stem structural characteristics based on spectra, which could facilitate the improvement of lodging resistance in crop breeding.

**Supplementary Information:**

The online version contains supplementary material available at 10.1186/s12870-025-06396-y.

## Background

Lodging is a common issue in cereal crops, and it can be categorized into two types: stem lodging and root lodging [1]. Stem lodging refers to the bending or breakage of the stem base due to mechanical failure [2]. There are different levels associated with stem characteristics such as morphology, anatomy, and cell wall composition and studies have found that stem structural characteristics are closely related to the lodging resistance of crops [1, 3]. Previous studies have shown that stem structural characteristics, such as vascular bundles and stem diameter, are significantly correlated with lodging resistance [4, 5]. Sorghum (*Sorghum bicolor* L.) is the fifth-ranking cereal crop worldwide and lodging is a serious challenge in sorghum production [6, 7]. Therefore, dissection of the stem structure is of great importance in sorghum.

The stem structure characteristics influence stem strength in sorghum. For example, the number of vascular bundles is positively correlated with breaking force [8]. However, traditional methods for determining stem structural characteristics are both complex and time-consuming. Firstly, the stem cross-section slices should dip into phloroglucinol [ethyl alcohol: water = 95:5] and concentrated hydrochloric acid [8] or placed in a fixing solution (70% ethanol: 5% acetic acid: 3.7% formaldehyde) for 24 h and then prepared for paraffin sectioning which needs dehydration in graded ethanol solutions [9]. Then, the stem slices are photographed with the microscope and measured using the Adobe Photoshop software [10]. Recently, near-infrared spectra (NIR) or visible and near-infrared spectra (VIS/NIR), combined with chemometrics has been an effective tool in the rapid determination of chemical components such as puerarin in *Puerariae Lobatae Radix*, leaf chlorophyll content in *Sassafras tzumu*, chrysin, and galangin in *Chinese propolis* [11–13]. Nevertheless, most NIR or VIS/NIR models for stem/stalk focus on the prediction of cell wall components [13–17], and less has been done on stem structural characteristics (such as the number and the area of vascular bundles). Plant cell walls are composed of carbohydrates such as cellulose and hemicellulose, which exhibit compositional differences among plants and different cells and tissues of a single plant [18]. For example, lignin is reported to be mainly distributed in the cell walls of the epidermis, mechanical tissue, xylem, and vascular bundle sheath in the sorghum stem [19]. Recently, several studies have reported that Near-Infrared (NIR) spectroscopy can be used to estimate the ratio of vascular bundles to parenchyma tissue in oil palm, distinguish stem regions in wheat, and predict the lodging index and stem breaking force in barley [20–22]. However, these studies are limited by small sample sizes and the low efficiency of phenotyping. Sorghum plant stems anatomical characteristics such as the number of vascular bundles and area of vascular bundles are crucial characteristics for determining stem lodging resistance, while methods for rapid determination of these traits are urgently needed in sorghum.

In this study, we attempted to establish VIS/NIR models for rapid determination of the stem anatomical parameters (area of large vascular bundles, area of small vascular bundles, number of large vascular bundles, number of small vascular bundles, stem area, stem circumference, rind area, rind thickness) in sorghum. Using two kinds of biomass powders (dry and water-washed) from 103 sorghum accessions with high diversity, the best combination of spectral pre-treatment and regression models was obtained. As a result, the VIS/NIR models established in our study could improve the efficiency of the relevant trait investigation for stem lodging resistance in sorghum.

## Results

### The high diversity of stem structural characteristics observed in sorghum accessions

In this study, 103 sorghum accessions collected from different regions (China, America, and India) were used for stem structural characteristics measurement and visible and near-infrared spectra acquisition (VIS/NIR) (Table S1). Description of the stem structural characteristics showed that the area of large/small vascular bundles (ALVB, ASVB), number of large/small vascular bundles (NLVB, NSVB), stem area (SA), stem circumference (SC), rind area (RA), and rind thickness (RT) exhibited greater variation such that the coefficient of variation (CV) of eight stem structural characteristics related traits was observed to be higher than 20% and ASVB showed the highest CV (60%) (Table 1).

**Table 1:** Descriptions of cell wall polymer features and stem structural characteristics in sorghum accessions

Trait	Mean	Min	Max	Range	SD	CV (%)	Skewness	Kurtosis
Area of LVB (ALVB, mm^2^)	0.0054	0.0019	0.0187	0.0168	0.0028	51.93	1.78	4.51
Area of SVB (ASVB, mm^2^)	0.002	0.0004	0.0062	0.0058	0.0011	55.99	1.44	3.22
Number of LVB (NLVB)	127.2	44	349	305	59.77	46.99	1.46	2.75
Number of SVB (NSVB)	135.3	39	309	270	48.76	36.04	1.12	1.99
Stem area (SA, mm^2^)	10.34	2.18	18.71	16.53	3.881	37.54	0.06	−0.70
Stem circumference (mm)	18.59	9.3	28.05	18.75	4.675	25.14	0.20	−0.85
Rind area (mm^2^)	0.6575	0.12	2.1	2.0	0.31	47.77	1.21	3.03
Rind thickness (mm)	0.093	0.04	0.26	0.22	0.034	36.53	1.78	4.96

**Fig. 1:**
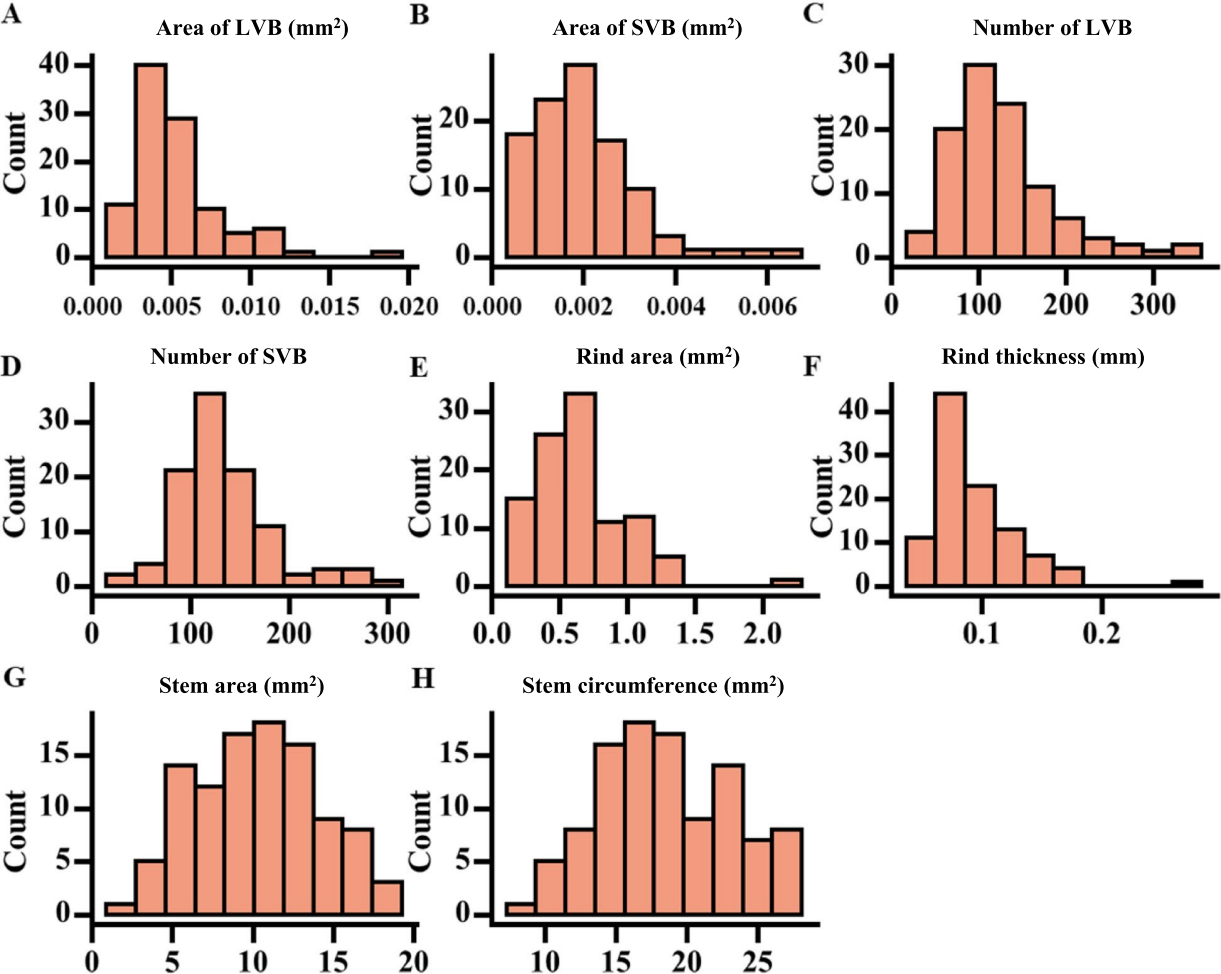
Distribution of stem structural characteristics in the sorghum population. **A** Area of large vascular bundles. **B** Area of small vascular bundles. **C** Number of large vascular bundles. **D** Number of small vascular bundles. **E** Rind area. **F** Rind thickness. **G** Stem area. **H** Stem circumference

In addition, the absolute value of skewness and kurtosis of eight stem structural characteristics related traits indicated that these traits approximately followed a normal distribution (Table 1; Fig. 1) (i.e., skewness < 2, kurtosis < 7) [23, 24]. As a result, the approximately normal distribution and greater variation of the stem structural characteristics are beneficial for model building.

**Fig. 2:**
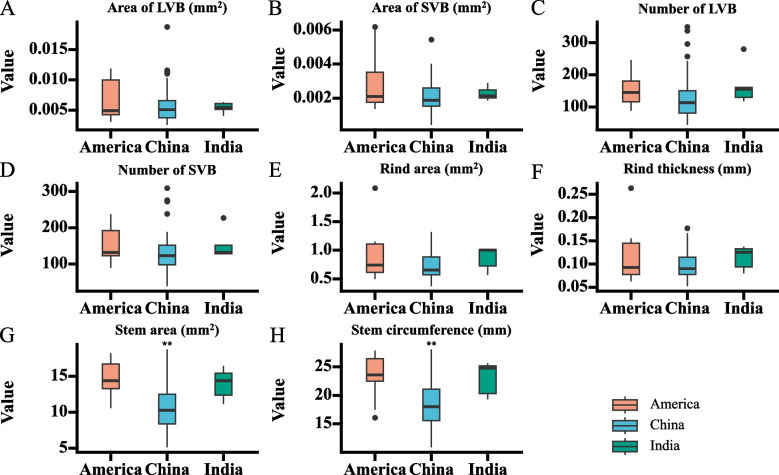
Comparison of stem structural characteristics among different counties. **A** Area of large vascular bundles. **B** Area of small vascular bundles. **C** Number of large vascular bundles. **D** Number of small vascular bundles. **E** Rind area. **F** Rind thickness. **G** Stem area. **H** Stem circumference. *P < 0.05, ** *P* < 0.01

Besides, a comparison of stem structural characteristics among different countries was conducted. As a result, we found that the stem area and stem circumference in China are significantly lower than in America and there are no differences in stem structural characteristics between India and America (Fig. 2). In addition, several accessions (F6, F7, and L34) had about 300 large vascular bundles while accessions SC34, F2, and F7 had about 300 small vascular bundles.

### Systematic integrated analysis revealed the correlation among stem structural characteristics, lodging index, and agronomic traits in sorghum

**Fig. 3:**
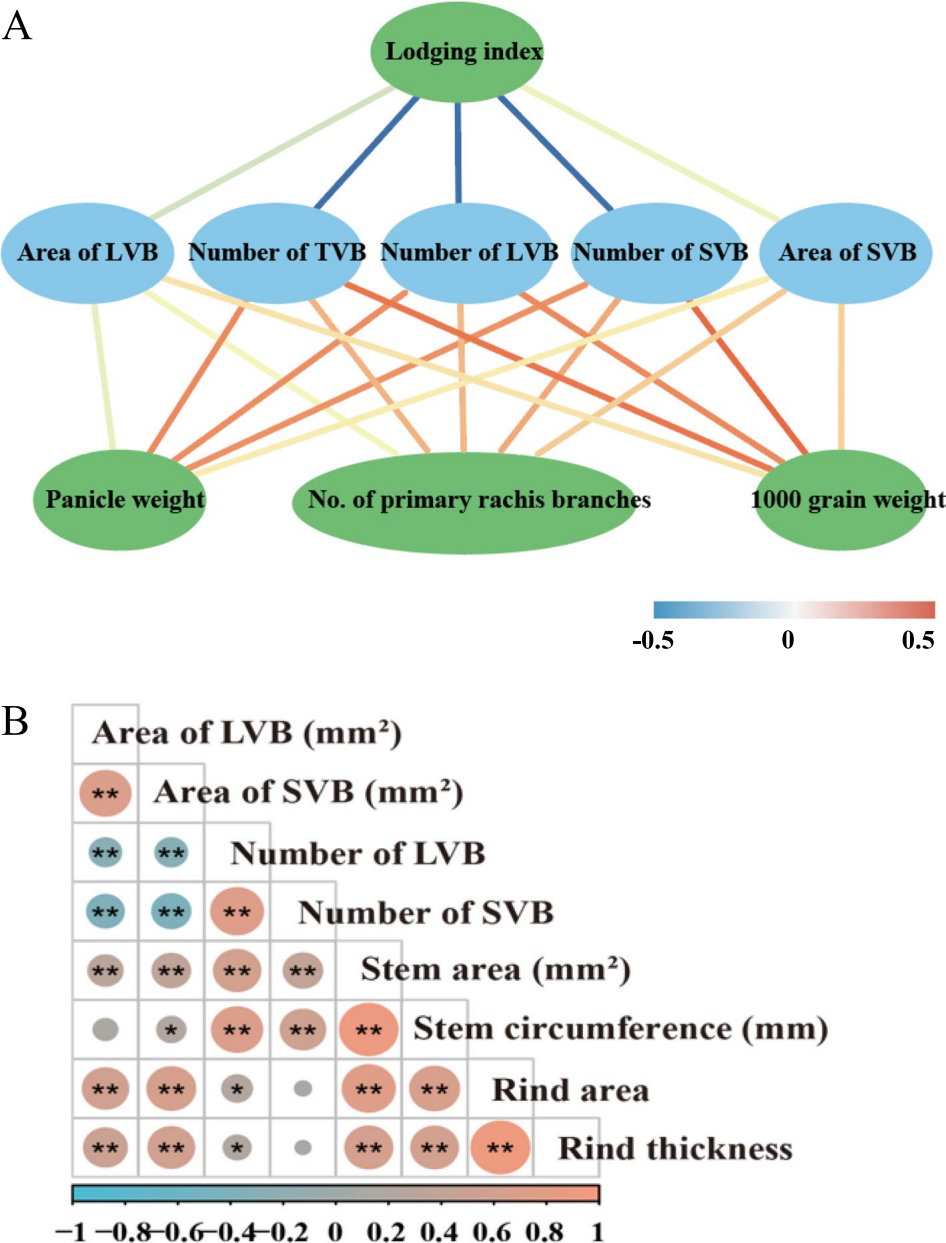
Correlation between stem characteristics and agronomic traits. **A** Correlation among lodging index, stem structural characteristics and agronomic traits. **B** Correlation among stem structural characteristics traits. *P < 0.05, ** *P* < 0.01

Systematically, correlation analysis was performed among stem structure, lodging index, and agronomic traits. The lodging index showed a significantly negative correlation with the number and area of vascular bundles. Detailly, the lodging index showed a higher correlation with the number of vascular bundles than the area of vascular bundles. (Fig. 3A). Besides, the number of vascular bundles showed a higher correlation with three agronomic traits (panicle weight, number of primary rachis branches, and 1000 grain weight) than the area of vascular bundles (Fig. 3A). Therefore, among stem structural-related traits, the number of vascular bundles played a crucial role in both stem lodging resistance and yield stability in sorghum. To better understand the stem structure, stem structural characteristics traits were correlated. Consequently, the number of LVB (NLVB), number of SVB (NSVB), stem area (SA), stem circumference (SC), rind area, and rind thickness showed significantly positive correlation with each other. In addition, ALVB and ASVB showed a significantly positive correlation with stem area, stem circumference, rind area, and rind thickness while it showed a negative correlation with the number of LVB (NLVB), and the number of SVB (NSVB) (Fig. 3B).

### Smooth spectrum after quality control and spectra pretreatments

**Fig. 4:**
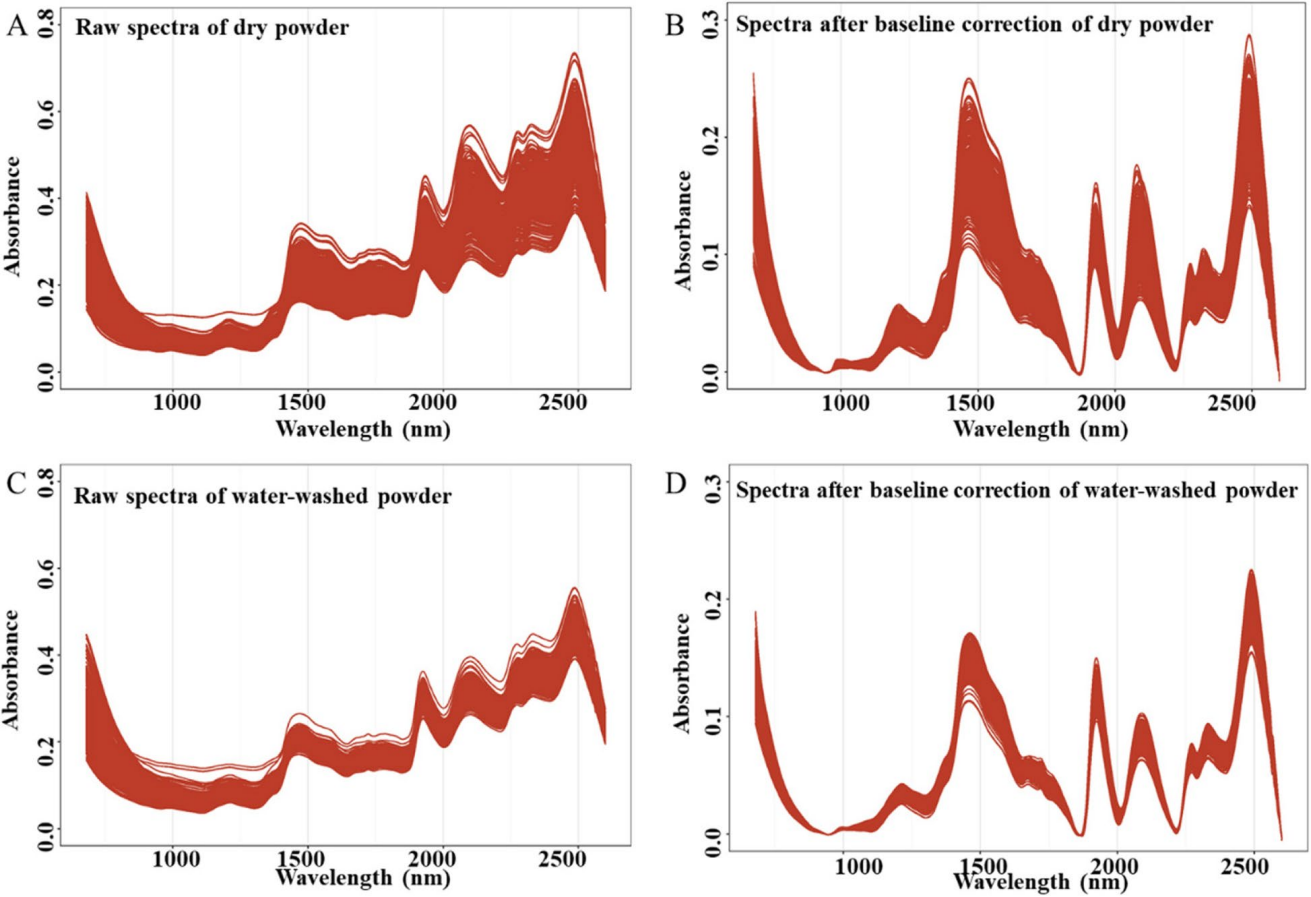
Raw and baseline corrected VIS/NIR spectra of dry and water-washed powder. **A** Raw spectra of dry powder; **B** Spectra after sample quality control and baseline correction of dry powder; **C** Raw spectra of water-washed powder; **D** Spectra after sample quality control and baseline correction of water-washed powder

From the raw spectra of dry and water-washed powder, we found that there were several outlier samples (Fig. 4A; C). Therefore, sample quality control was performed and twenty samples were deleted in both dry and water-washed powder (Fig. S1). Then, baseline processing was carried out for dry and water-washed powder (Fig. 4B; D). After quality control and spectra correction, the spectra of dry and water-washed powder were smooth and adequate for further modeling.

### The combination of MSC pretreatment and SVR model was the best in the prediction of stem structural characteristics traits

**Fig. 5:**
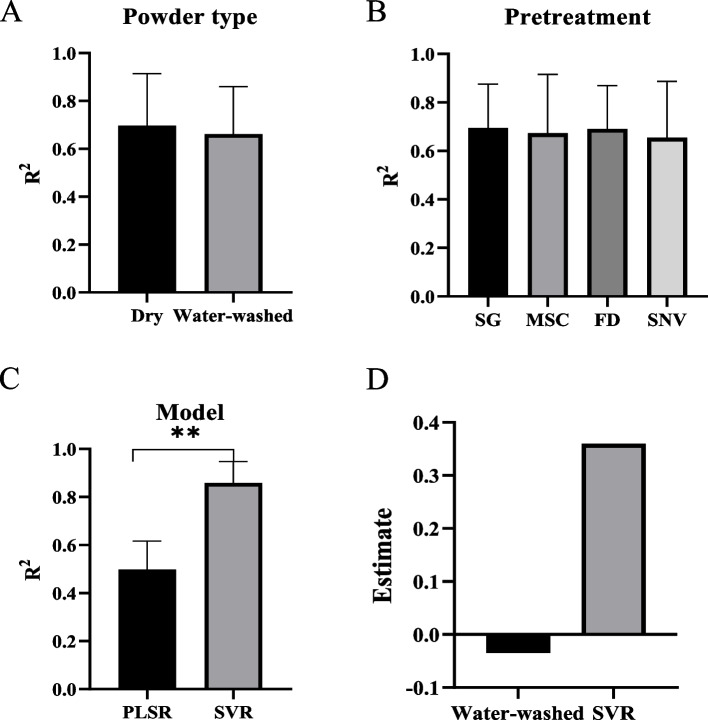
Comparison of powder type, model, and pretreatment of spectra in the prediction of stem structural characteristics in sorghum. **A** Comparison of powder type in the prediction of stem structural characteristics. **B** Comparison of the pretreatment in the prediction of stem structural characteristics. **C** Comparison of the model in the prediction of stem structural characteristics. **D** Regression analysis of different factors in the prediction of stem structural characteristics. *P < 0.05, ** < 0.01

**Fig. 6:**
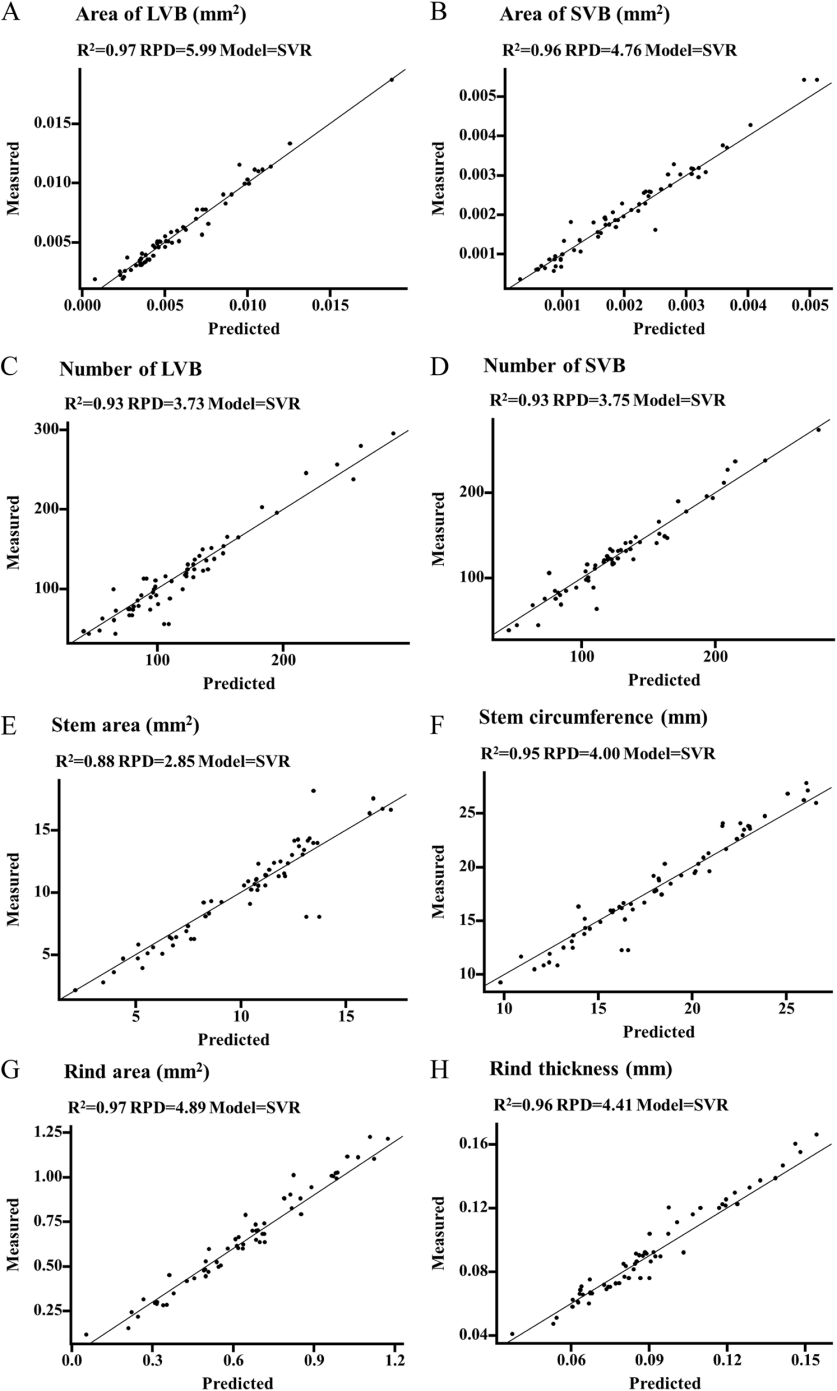
Comparison of predicted and measured values of stem structural characteristics by the combination (dry + SNV + SVR). **A** Area of LVB. **B** Area of SVB. **C** Number of LVB. **D** Number of SVB. **E** Stem area. **F** Stem circumference. **G** Rind area (mm.^2^). H: Rind thickness (mm)

As the cell wall polymer features could be precisely predicted by the VIS/NIR models, the prediction of stem structural characteristics was used. As a result, we found that the dry powder and SVR model had a higher coefficient of determination (*R*-squared) than the water-washed powder and PLSR model while *R*^2^ showed no significant difference among four pretreatments of spectra (Table S2; Fig. 5A-C). Detailly, the *R*^2^ and relative percent difference (RPD) of the combination (dry + MSC + SVR) for prediction of stem structural characteristics ranged from 0.88 – 0.97, and 2.85 – 5.99 in validation data, respectively (Fig. 6). The *R*^2^ and RPD of stem area (0.88, 2.85) was the lowest while *R*^2^ and RPD of other traits were over 0.93 and 3.73.

Besides, we conducted a regression analysis of the powder type, spectra pretreatments, and model to examine the effect on *R*^2^. As a result, the SVR model showed a significant positive effect on the accuracy of the prediction for stem structural characteristics related traits and the water-washed powder had significant negative effects (Fig. 5D). The comprehensive results suggested that the combination (dry + MSC + SVR) had good prediction capacity, which could be applicable for the prediction of stem structural characteristics traits. Summarily, the SVR model is the most important positive factor in predicting stem structural characteristics and the combination (dry + MSC + SVR) was the ideal model for the prediction of stem structural characteristics related traits.

## Discussion

The internal structure of stems has been reported to play important roles in stem lodging resistance and yield stability in cereal crops such as sorghum, wheat, and rice [8, 25, 26]. In detail, the number of vascular bundles and the area of vascular bundles were positively associated with the lodging resistance in sorghum [8], wheat [3], and rice [4]. Besides, the vascular bundles also regulate the long-distance translocation of photoassimilates from source to sink tissues through their structural characteristics (bundle number, size, developmental stage) and functional transport capacity [27]. However, the measurement of these traits is laborious and thus less investigated in sorghum. Moreover, the roles of stem characteristics in lodging resistance and agronomic traits are poorly understood. In this study, the optimized VIS/NIR models for the high-throughput prediction of stem structural characteristics were established. The results allowed us to give novel insight into the effects of stem-relevant traits on sorghum lodging resistance and agronomic traits.

In this study, we found that the model types were the major factor in determining the prediction performance and SVR was the most significant positive factor for the prediction of stem structural characteristics. In addition, we compared two different types of powders (dry and water-washed) in VIS/NIR spectra acquisition and found that the dry powder showed higher R^2^ than water-washed powder in the VIS/NIR model. This might be due to the alteration in the polymorphs and crystallinities of cellulose during the hydrothermal treatment of hot water as reported by previous studies [28]. So, the dry biomass was recommended for direct VIS/NIR spectra acquisition. In addition, overfitting is a potential concern for the VIS/NIR model for predicting sorghum stem structure. Firstly, the samples utilized in this study were gathered from a single environment, resulting in a lack of environmental diversity. This limitation implies that the stem components may not exhibit stability across varying conditions. Environmental factors—such as temperature, management practices, and water availability—could significantly influence stem structure traits. Second, the relatively small sample size represents another contributing factor to potential overfitting. With only a limited number of sorghum accessions, the full spectrum of variation within sorghum may not be adequately represented. Thus, by integrating a diverse range of sorghum accessions across varied environments, the model can be enhanced to develop a robust VIS/NIR model for accurately predicting stem structure in sorghum.

In this study, we found that plant stem structural characteristics, especially the number of vascular bundles were useful in improving plant lodging resistance. In our previous studies, we have developed a semi-automatic high-throughput method for the phenotyping cross-sectional morphology of sorghum stems [8]. In this study, we build VIS/NIR models for the prediction of stem structural characteristics in sorghum. It is foreseeable that the high performance of prediction of stem structural characteristics is based on the significant internal association between cell wall polymer features and stem structural characteristics-related traits. In addition, the accurate and rapid prediction of the stem-associated internal traits in sorghum could broaden VIS/NIR usage for cell wall components, stem structure, and lodging index prediction. The high performance of the VIS/NIR model for stem structural characteristics could facilitate rapid analysis of the stem lodging-related traits in sorghum.

## Conclusion

In this study, precise and high throughout VIS/NIR models were obtained for the prediction of stem structural characteristics in sorghum. Importantly, the established models for the prediction of these stem-associated internal traits would improve the efficiency of plant lodging assessment in sorghum.

## Materials and methods

### Plant materials for stem structural characteristics

**Fig. 7:**
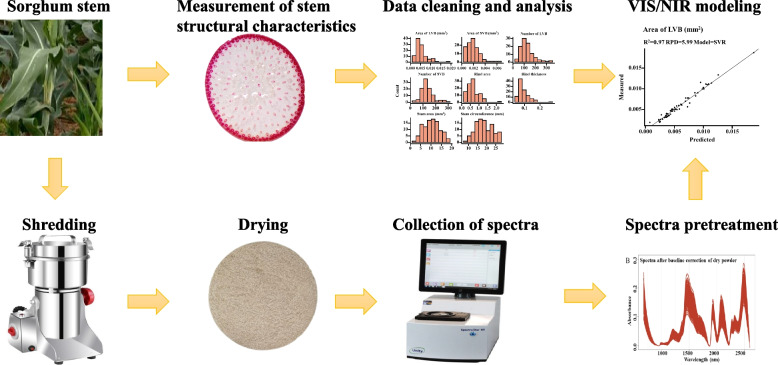
The pipeline for data collection and data analysis

The sorghum set included 103 accessions from a mini-core collection and breeding varieties from China, America, and India. These accessions were planted in the experimental field on the campus of Guangxi University (Nanning, China, 22°48′ N, 108°22′ E) in 2021. Twenty individuals from each genotype were grown in two rows with a distance of 30 cm between plants in each row and 60 cm between rows. Field management essentially followed the local sorghum cropping practices. The lines were harvested individually at maturity to prevent seed contamination among lines. The harvested stalk was dried at 105℃ for 20 min and then dried at 60℃ for three days (Fig. 7). Afterward, the stalks were milled using a grinder (RS-FS1406) and filtered with a 40-mesh sieve. The powder was packed in bags, dried at 60℃ until constant weight, and maintained in boxes with a silica gel dryer. Furthermore, the bag was washed and treated at 105℃ until constant weight. The constant weight was regarded as a structural carbohydrate and the powder was named water-washed powder. The stem cross-section slices were collected from the sorghum at the booting stage and the stem structural characteristics were obtained by a semi-automatic high-throughput method [8].

### Collection and processing of the VIS/NIR spectra

The VIS/NIR spectra were collected by Spectra Star 2600XT-R (UNITY SCIENTIFIC) at Huazhong Agricultural University. The wavelength of the spectra ranged from 680 to 2600 nm with a resolution of 1 nm and instrument calibration is performed at regular intervals to ensure consistency of measurement results. Both dry and water-washed powder were used for spectra collection and each sample was repeatedly loaded and scanned three times. Then, sample quality control was performed by isolation forest by the R package isotree (version 0.5.22) (https://github.com/david-cortes/isotree). Besides, to filter the noise brought by various factors and obtain a better performance model, standard normal variation (SNV), multiple scatter correction (MSC), first derivative (FD), and Savitzky-Golay (SG) were used after the baseline correction for dry (Fig. S2) and water-washed powder (Fig. S3), respectively.

### VIS/NIR data calibration

Due to many factors (such as instrument drift, sample inhomogeneity, etc.) that could bring baseline drift problems in the near-infrared spectra and may interfere with our accurate identification and quantification of compounds in the sample, raw spectra should be corrected for further analysis. Baseline correction was carried out to improve the efficiency of the model. In addition, standard normal variation (SNV), multiple scatter correction (MSC), first derivative (FD), and Savitzky-Golay (SG) were performed after baseline correction, respectively by R package pls (version 2.8–1) and prospectr (version 0.2.7) (https://github.com/l-ramirez-lopez/prospectr). The combination of different pretreatment of spectra was used to reduce the noise and increase the accuracy of the models.

As for modeling, 103 sorghum accessions were separated into calibration data (80%) and validate data (20%) by the KenStone function by R package prospectr (https://github.com/l-ramirez-lopez/prospectr). To obtain a robust model, supported vector machine regression (SVR), and partial least square regression (PLSR) were used for stem structural characteristics prediction based on the spectra. For each SVR, a grid search was performed to search for the best gamma and cost. The data arrangement, model optimization, and data visualization were performed in R software. Based on the output of the PLSR and SVR model, the coefficient of determination (*R*^2^), and residual predictive deviation (RPD) were used to evaluate the performance of these models [29].

### Quick measurement of stem structural characteristics

The basal second internode stem was cut with a thickness of 0.2 ~ 0.4 mm at the booting stage, and the samples were treated with 5% phloroglucinol and concentrated stain with hydrochloric acid for 15 s, and the stem structure was photographed by OLYMPUSSZ61 stereomicroscope with a magnification of 6.7 × to 15 × , and pictures were taken for further use. Then, software “Labelme” was used to label the features such as vascular bundles of saved pictures, and the labeled pictures were saved as JSON files. The JSON format file was used to extract stem structural characteristics by Python (3.9.2) [30].

## Supplementary Information

Additional file 1: Table S1. Region and stem structural characteristics of sorghum accessions; Table S2. Statistics for prediction of stem structural characteristics in sorghum.

Additional file 2: Fig. S1. Sample quality control and dataset partitioning. A: Diagnosis of bad samples of dry powder; B: Diagnosis of bad samples of water-washed powder; C: Dataset partitioning of dry powder (trait: area of LVB under MSC pretreatment); D: Dataset partitioning of water-washed powder (trait: area of LVB under MSC pretreatment). Fig. S2. Pretreatments of spectra of dry powder. A: Spectra of SNV (standard normal variation) treatment; B: Spectra of SG (Savitzky-Golay) treatment; C: Spectra of FD (first derivative) treatment; D: Spectra of MSC (multiple scatter correction) treatment. Fig. S3. Pretreatments of spectra of water-washed powder. A: Spectra of SNV (standard normal variation) treatment; B: Spectra of SG (Savitzky-Golay) treatment; C: Spectra of FD (first derivative) treatment; D: Spectra of MSC (multiple scatter correction) treatment.

## Data Availability

Data is provided within the manuscript or supplementary information files. Besides, NIR spectra data will be made available on request. The data that support the findings of this study are available from the first author (15,611,821,765@163.com).
