# Supplementary material for: A precise and high-throughput assay for stem structural characteristics deepens understanding of lodging resistance in sorghum
Source: BMC Plant Biol. 2025 Mar 27;25:386. doi: 10.1186/s12870-025-06396-y (PMC11948900; doi:10.1186/s12870-025-06396-y)
Supplement: Supplementary file 2 — Additional file 2: Fig. S1. Sample quality control and dataset partitioning. A: Diagnosis of bad samples of dry powder; B: Diagnosis of bad samples of water-washed powder; C: Dataset partitioning of dry powder (trait: area of LVB under MSC pretreatment); D: Dataset partitioning of water-washed powder (trait: area of LVB under MSC pretreatment). Fig. S2. Pretreatments of spectra of dry powder. A: Spectra of SNV (standard normal variation) treatment; B: Spectra of SG (Savitzky-Golay) treatment; C: Spectra of FD (first derivative) treatment; D: Spectra of MSC (multiple scatter correction) treatment. Fig. S3. Pretreatments of spectra of water-washed powder. A: Spectra of SNV (standard normal variation) treatment; B: Spectra of SG (Savitzky-Golay) treatment; C: Spectra of FD (first derivative) treatment; D: Spectra of MSC (multiple scatter correction) treatment. [file 12870_2025_6396_MOESM2_ESM.pptx]

## Slide 1
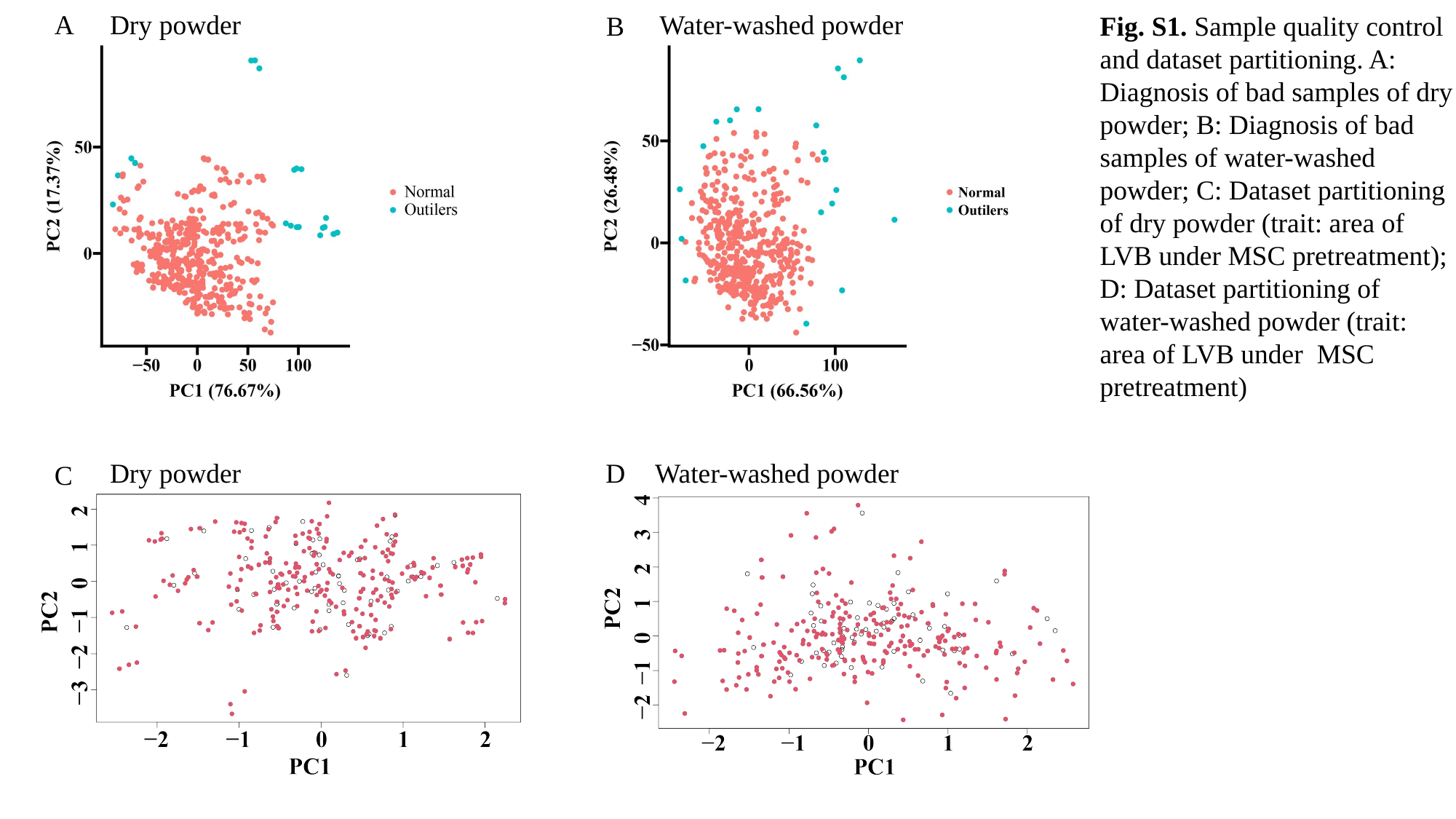

A
Dry powder
Water-washed powder
B
Dry powder
D
Water-washed powder
C
Fig. S1. Sample quality control and dataset partitioning. A: Diagnosis of bad samples of dry powder; B: Diagnosis of bad samples of water-washed powder; C: Dataset partitioning of dry powder (trait: area of LVB under MSC pretreatment); D: Dataset partitioning of water-washed powder (trait: area of LVB under MSC pretreatment)

## Slide 2
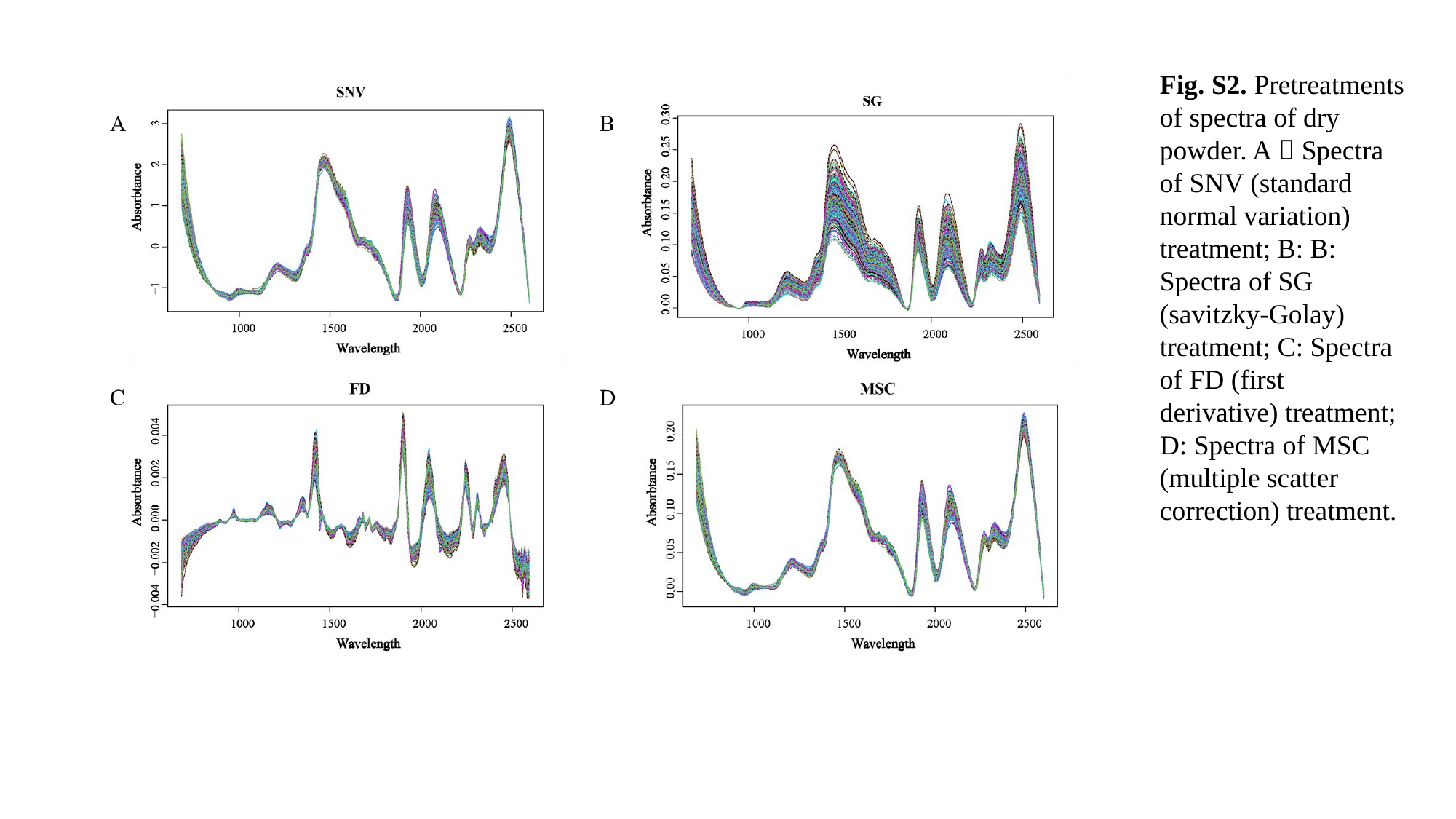

Fig. S2. Pretreatments of spectra of dry powder. A：Spectra of SNV (standard normal variation) treatment; B: B: Spectra of SG (savitzky-Golay) treatment; C: Spectra of FD (first derivative) treatment; D: Spectra of MSC (multiple scatter correction) treatment.

## Slide 3
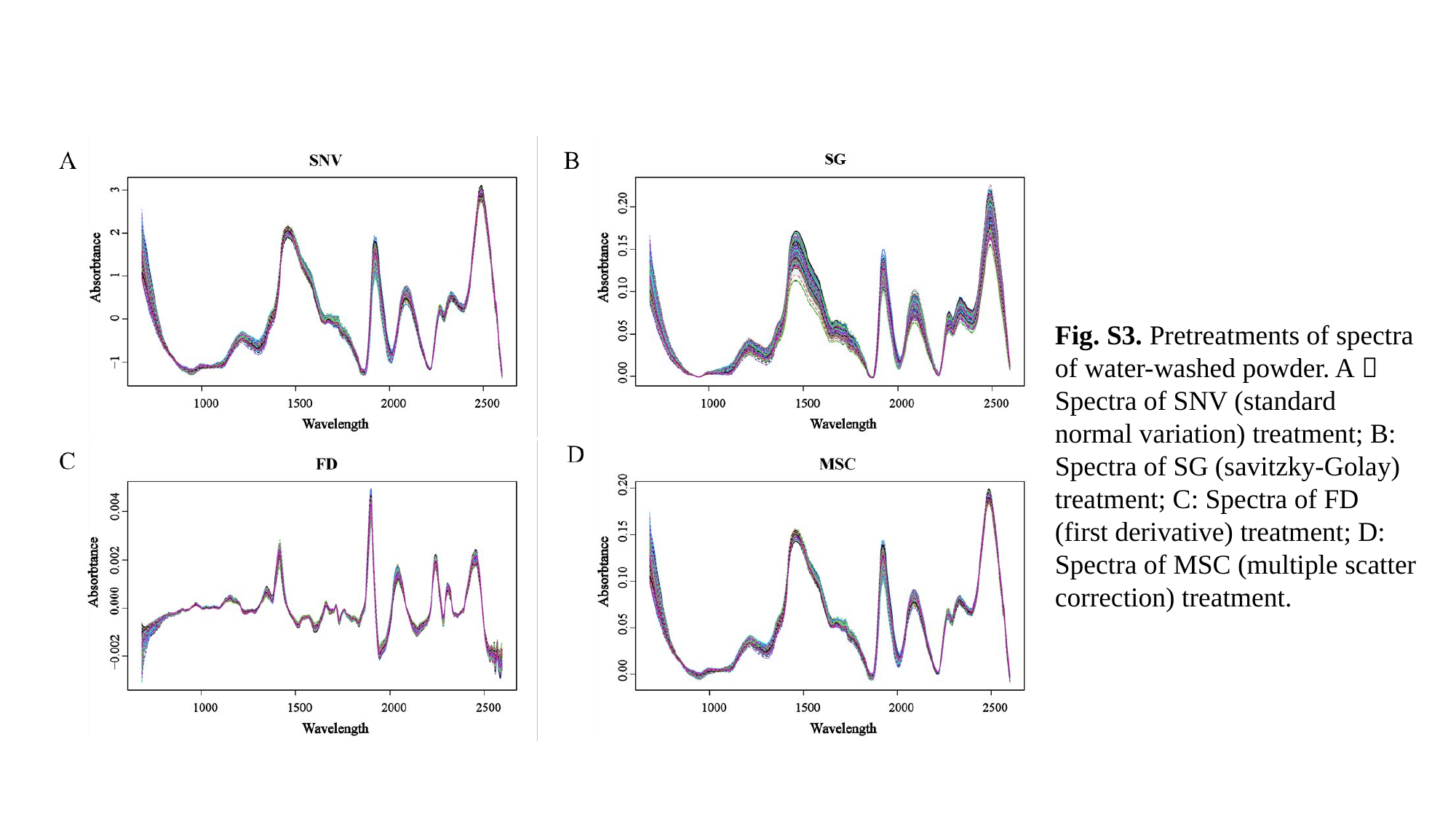

Fig. S3. Pretreatments of spectra of water-washed powder. A：Spectra of SNV (standard normal variation) treatment; B: Spectra of SG (savitzky-Golay) treatment; C: Spectra of FD (first derivative) treatment; D: Spectra of MSC (multiple scatter correction) treatment.
